# Exhaled Breath Metabolomics for the Diagnosis of Pneumonia in Intubated and Mechanically-Ventilated Intensive Care Unit (ICU)-Patients

**DOI:** 10.3390/ijms18020449

**Published:** 2017-02-19

**Authors:** Pouline M. P. van Oort, Sanne de Bruin, Hans Weda, Hugo H. Knobel, Marcus J. Schultz, Lieuwe D. Bos

**Affiliations:** 1 Department of Intensive Care, Academic Medical Centre, Meibergdreef 9, 1105 AZ Amsterdam, The Netherlands; pouline.vanoort@gmail.com (P.M.P.v.O.); sanne_debruin@hotmail.com (S.d.B.); marcus.j.schultz@gmail.com (M.J.S.); lieuwe.bos@gmail.com (L.D.B.); 2 Philips Research, 5656 AE Eindhoven, The Netherlands; hans.weda@philips.com (H.W.); hugo.knobel@philips.com (H.H.K.)

**Keywords:** respiratory infection, breathomics, mechanical ventilation, intensive care, critical care, diagnosis, volatile organic compounds

## Abstract

The diagnosis of hospital-acquired pneumonia remains challenging. We hypothesized that analysis of volatile organic compounds (VOCs) in exhaled breath could be used to diagnose pneumonia or the presence of pathogens in the respiratory tract in intubated and mechanically-ventilated intensive care unit patients. In this prospective, single-centre, cross-sectional cohort study breath from mechanically ventilated patients was analysed using gas chromatography-mass spectrometry. Potentially relevant VOCs were selected with a *p*-value < 0.05 and an area under the receiver operating characteristics curve (AUROC) above 0.7. These VOCs were used for principal component analysis and partial least square discriminant analysis (PLS-DA). AUROC was used as a measure of accuracy. Ninety-three patients were included in the study. Twelve of 145 identified VOCs were significantly altered in patients with pneumonia compared to controls. In colonized patients, 52 VOCs were significantly different. Partial least square discriminant analysis classified patients with modest accuracy (AUROC: 0.73 (95% confidence interval (CI): 0.57–0.88) after leave-one-out cross-validation). For determining the colonization status of patients, the model had an AUROC of 0.69 (95% CI: 0.57–0.82) after leave-one-out cross-validation. To conclude, exhaled breath analysis can be used to discriminate pneumonia from controls with a modest to good accuracy. Furthermore breath profiling could be used to predict the presence and absence of pathogens in the respiratory tract. These findings need to be validated externally.

## 1. Introduction

Severe community- and hospital-acquired pneumonia (CAP and HAP) represent a major clinical problem associated with a high mortality, and frequently requires admission to the intensive care unit (ICU), intubation, and mechanical ventilation [1]. The diagnosis of CAP and HAP is currently based on clinical, radiological, and microbiological criteria, but these criteria have several disadvantages [2]. Physical examination has a high inter-observer variability and a moderate sensitivity and specificity [3]. Chest X-ray has a poor sensitivity and positive predictive value for CAP and HAP [4]. Bacterial cultures need several days before showing growth and results could be false-negative due to previously-administered antibiotics [5]. An ideal diagnostic test would be objective, non-invasive, and clinically available at the bedside, enabling rapid exclusion of the presence of pneumonia and, thus, withholding certain patients from receiving antimicrobial treatment unnecessarily. 

Exhaled breath contains metabolites in the gas phase called volatile organic compounds (VOCs) that are produced by the host and bacteria. Different bacterial strains show distinct patterns of VOCs in vitro and in animal models [6,7,8,9,10,11,12].Therefore, exhaled breath analysis might be used to identify the causative pathogen in patients suspected of CAP/HAP [13]. A recent study shows that exhaled breath analysis can discriminate between VOC profiles of patients with a high risk of developing nosocomial pneumonia with and without a significant pathogen load in the lower respiratory tract [14]. Thermal desorption with gas chromatography coupled to mass spectrometry (TD-GC-MS) can be used to separate, quantify, and identify VOCs [15].

The aim of this study was to determine which VOCs could be used to identify patients with CAP or HAP using GC-MS. Our data suggest that VOCs in exhaled breath can be used to discriminate between intubated and mechanically-ventilated patients in ICU with CAP or HAP and ventilated patients without pneumonia with moderate accuracy. 1-Propanol was found to be consistently lower in patients with pneumonia and, independently, also in patients with colonized airways, and might be a marker for bacterial presence and growth.

## 2. Results

### 2.1. Subjects

**Figure 1:**
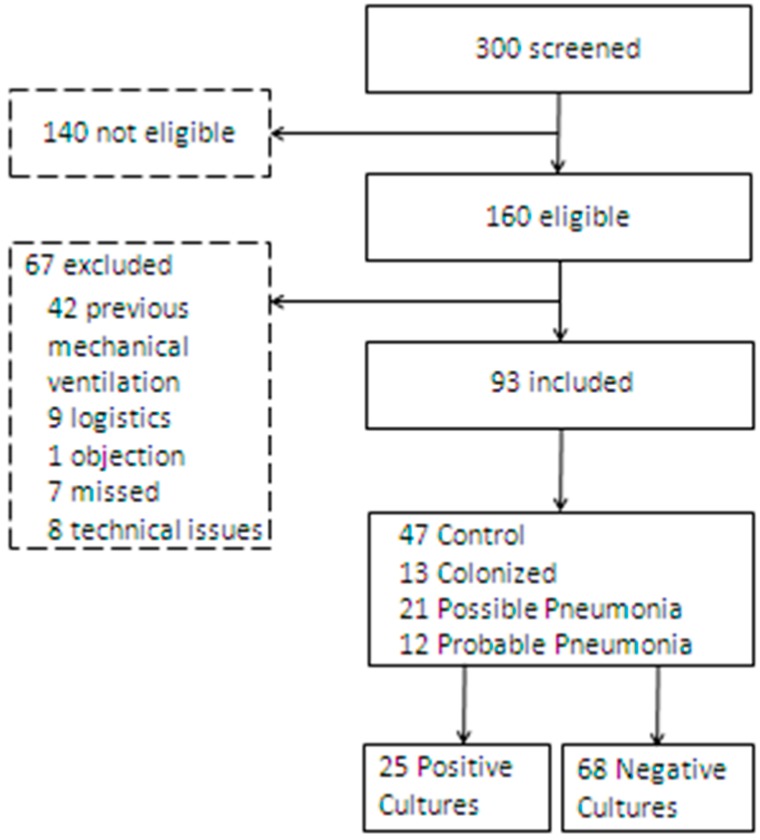
Flowchart of screened patients.

A total of 300 patients were screened (Figure 1), of whom 160 were eligible. Sixty-seven patients were excluded for several reasons, e.g., previous mechanical ventilation or technical issues. Finally, 93 patients were included. Twelve patients (13%) had probable pneumonia and were considered cases. Forty-seven patients (50%) were not suspected of pneumonia and did not have colonized airways, and were included as controls. Twenty-one patients had a possible pneumonia (23%), and 13 patients who were not suspected of pneumonia, but had colonized airways. In total, 25 (27%) patients had positive cultures, irrespective of the suspicion of pneumonia. The baseline demographic and clinical characteristics of the study population are shown in Table 1.

### 2.2. Probable Pneumonia vs. Controls (Patients without Pneumonia and without Colonized Airways)

**Figure 2:**
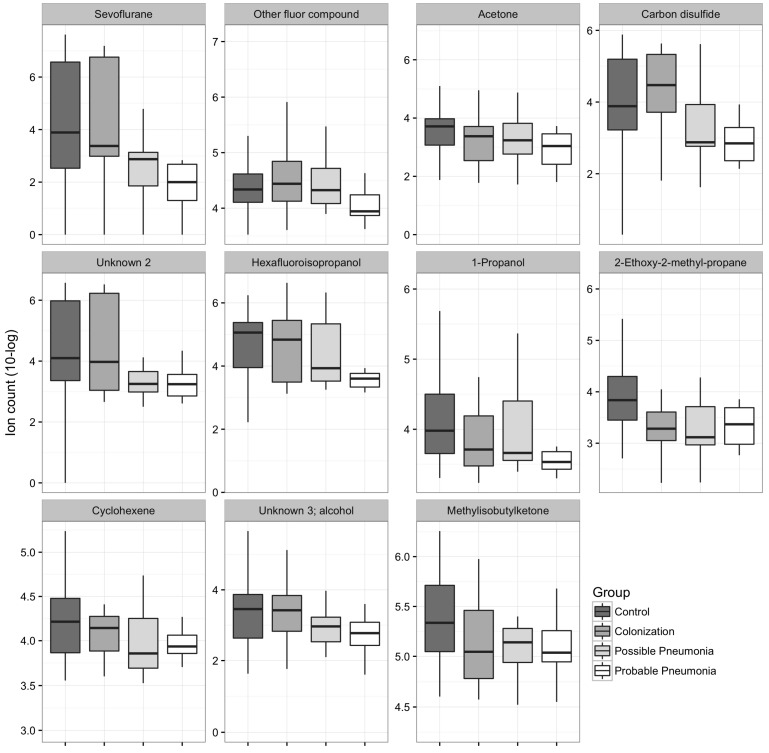
Ion count of volatile organic compounds (VOCs) in all four groups that showed a *p*-value < 0.05 between patients with probable pneumonia compared to controls.

**Figure 3:**
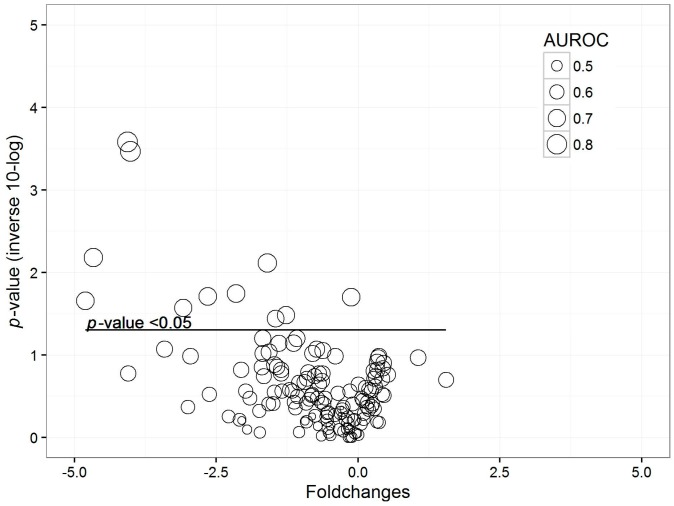
Volcano plot for comparison of patients with probable/proven pneumonia vs. controls. Each dot represents a VOC. The *y*-axis shows the inverse of the 10-log transformed *p*-value: the higher on the axis, the more significant. The *x*-axis shows the fold change between the groups. The size of the dots represents the AUROC.

One hundred forty-five VOCs were found in the breath of all patients. Concentrations of eleven (7.6%) VOCs were significantly lower in the breath of cases than in that of controls (*p*-value < 0.05; see Figure 2 for distribution and names). These results were visualized in a volcano plot (Figure 3). Ten of these VOCs also showed an area under the receiver operating characteristics curve (AUROC) higher than 0.7. 1-Propanol and hexafluoroisopropanol showed the highest AUROC of, respectively, 0.83 (CI 0.72–0.93) and 0.82 (CI 0.72–0.93). One thousand permutations of the labels were performed and 1.7% and 2.3% of these random scenarios resulted in a similar or better *p-*value and AUROC, respectively.

**Figure 4:**
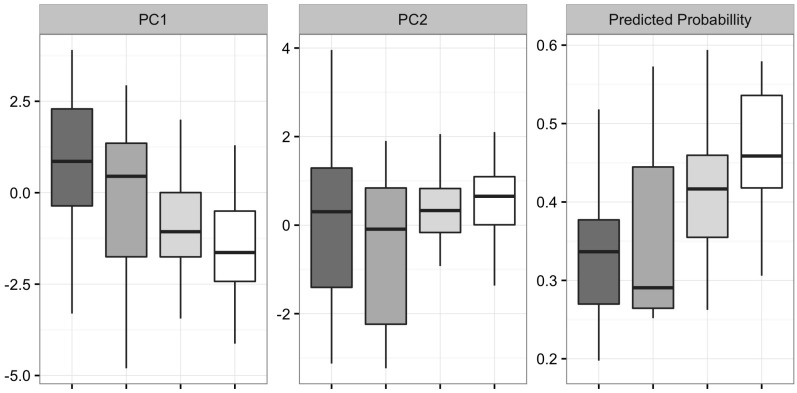
First (PC1) and second (PC2) principal component explained 35.1% and 22.4% of the variance, respectively. Predicted probability calculated by the PLSDA model. From left to right: controls, colonized controls, possible pneumonia, and probable pneumonia.

Principal component analysis showed a significantly lower first principal component score (explaining 35.1% of variance) for patients with probable pneumonia (*p* < 0.001; see also Figure 4). The second principle component (22.4% of variance) did not show significant differences (*p* = 0.43) between cases and controls. Partial least squares discriminant analysis (PLSDA) was used to classify cases and controls (Table 2). The AUROC for the PLSDA model was 0.87 (95% CI: 0.75–0.98) for in-set analysis and 0.73 (95% CI: 0.57–0.88) after leave-one-out cross-validation. Prediction of pneumonia probability in patients with possible pneumonia and without pneumonia with colonized airways results gave results in between cases and controls (Figure 4).

### 2.3 Patients with Positive Cultures vs. Patients with Negative Cultures

**Figure 5:**
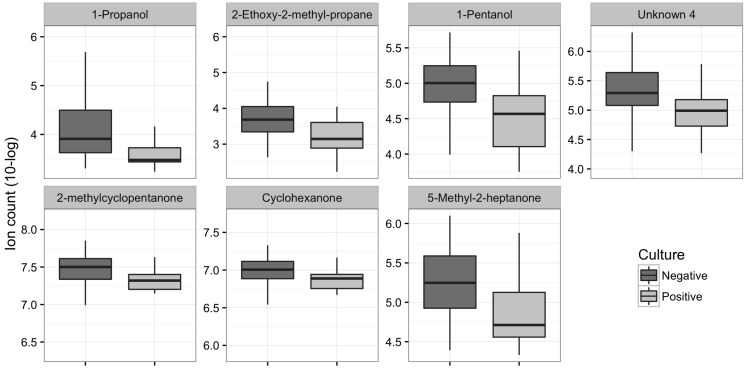
Ion count of VOCs that showed a *p-*value < 0.001.

**Figure 6:**
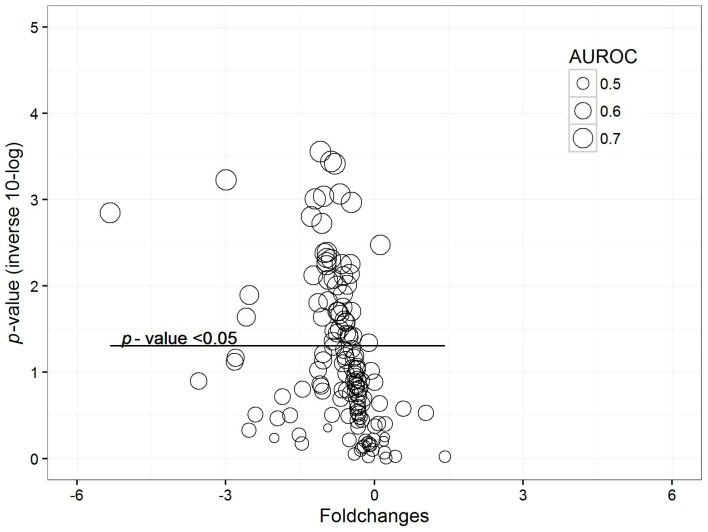
Volcano plot for comparison of patients with probable/proven pneumonia vs. controls. Each dot represents a VOC. The *y*-axis shows the inverse of the 10-log transformed *p*-value: the higher on the axis, the more significant. The *x*-axis shows the fold change between the groups. The size of the dots represents the AUROC. The horizontal line shows *p* = 0.05 with dots above this line having *p* < 0.05.

Concentrations of 52 VOCs (35.9%) were significantly lower in patients with colonized airways than in patients without colonization (*p*-value < 0.05). Seven out of these VOCs showed a *p*-value < 0.001 (Figure 5). Moreover, 11 out of the 52 VOCs showed an AUROC of above 0.7. These results were visualized in a volcano plot (Figure 6). One thousand permutations of the labels were performed and 1.4% and 0.06% of these random scenarios resulted in a similar or better *p-*value and AUROC, respectively.

Principal component analysis showed a significant higher first principal component score (explaining 62.5% of variance) for patients with colonized airways (*p* < 0.01). The AUROC for the PLSDA model was 0.79 (95% CI: 0.70–0.90) for in-set analysis and 0.69 (95% CI: 0.57–0.82) after leave-one-out cross-validation.

## 3. Discussion

The results of this study indicate that intubated and mechanically-ventilated ICU patients with and without pneumonia can be discriminated with moderate to good accuracy with exhaled breath analysis by GC-MS. Patients with colonized airways or with a low suspicion of pneumonia were classified as two separate groups. Airway colonization, irrespective of the likelihood of pneumonia, also resulted in a changed concentration of several VOCs in the exhaled breath.

We found a moderate to good accuracy with our models after leave-one-out cross-validation, but several other studies on breath analysis in pneumonia have reported higher diagnostic accuracies. Schnabel et al. reported an AUROC of 0.87 in diagnosing ventilator-associated pneumonia (VAP) [14]. All included patients in that study underwent a diagnostic bronchoalveolar lavage. Although the optimal diagnostic strategy for pneumonia is discussed [16,17,18,19,20], bronchoalveolar lavage is generally considered a better gold standard, and this may partly explain the higher accuracy that was found previously [21].

We found eleven VOCs (Figure 2) that we considered significant when distinguishing between patients with a probable pneumonia and controls (*p* < 0.05). Sevoflurane, hexafluoroisopropanol, and the other fluor compound are probably of an exogenous origin and could, thus, be regarded as falsely discovered. Acetone is generally present in high concentrations in breath and is also produced by most bacteria [13]. Carbon disulfide is a volatile liquid that is frequently used as a chemical or industrial solvent. 1-Propanol is most importantly produced by *Escherichia coli*, which might use this alcohol to hinder growth of other pathogens [13]. Propanes are normally used as fuels (e.g., for engines or residential central heating) and might, thus, be of false-discovery as well. Cyclohexene is a hydrocarbon that is used to fabricate other chemicals. The production of methyl ketones occurs during decarboxylation of fatty acid derivates and the longer 2-ketones have been described as classical biomarkers for *Pseudomonas aeruginosa* [13]. For certain compounds previously linked to pneumonia (e.g., aldehydes [13]) no significant difference was seen between patients with probable pneumonia and controls. All discriminative molecules were found in lower concentrations in patients with pneumonia compared to controls, as well as in colonized patients compared to non-colonized patients. This is a remarkable finding since most reported biomarkers (e.g., procalcitonin, C-reactive protein) increase during pneumonia [22]. Furthermore, this has not been reported in breath profile studies about respiratory tract infections before. To our knowledge no other studies about breath profiling have yet been performed in patients with CAP or HAP. However, a large number of studies have been conducted in patients with other inflammatory pulmonary diseases, including, but not limited to, asthma, chronic obstructive pulmonary disease (COPD), Acute Respiratory Distress Syndrome (ARDS), and ventilator-associated pneumonia (VAP) [23,24,25,26,27]. Hexanal is an example of a VOC that has been shown to have potential to discriminate between COPD patients and healthy controls [28], whereas nonanal is associated with tobacco consumption in healthy volunteers [29]. Results of another study comparing COPD patients and controls, showed that the VOCs that discriminated mostly appeared to be predominantly lower in the COPD patients [24]. We should be careful to extrapolate these results from chronic inflammation to acute illness, but it suggests that inflammation can lead to a decreased concentration of certain VOCs in exhaled breath. Schnabel et al. [14] also found some VOCs that were decreased in patients with VAP. Nevertheless, more than half of the discriminative VOCs were higher in patients with VAP compared to controls. The cause of decreased VOCs is yet unclear. We hypothesize that inflammation caused by pneumonia could lead to altered gas exchange over the lung–blood barrier, resulting in decreased VOC excretion. Alternatively, inflammatory or bacterial cells may use the VOCs or their metabolic precursor, resulting in a lower concentration in the exhaled breath. Furthermore, infection or colonization could alter the normal microbiome in the lower and upper respiratory tract due to inflammation, overgrowth of certain pathogens, or administration of antibiotics [30]. The decreased VOCs could reflect the suppression of the lung microbiome. Finally, one of the reasons that this study did not confirm that specific VOCs produced by bacteria increase during infection, could be that the significant changes found in this study were all part of the host response and less influenced by breath profiles from bacteria. Twelve patients were diagnosed with a probable pneumonia. For these patients we have found nine different pathogens. In other words, the frequency of each pathogen across the pneumonia group is too low. Each pathogen produces its own specific breath profile [12,31]. Due to the low frequency of each pathogen we lacked the statistical power to find significant VOC compositions produced by the bacteria, within the pneumonia group.

We found that the VOCs that discriminated between patients with pneumonia and controls, and between colonized and non-colonized airways, were different ones; only six out of 57 VOCs matched. 1-Propanol was the only VOC that was highly discriminatory in both analyses. Therefore, this is the only VOC identified in this study that might qualify as a biomarker. 1-Propanol differs most between patients with positive cultures and those with negative cultures, hence, primarily being a measure of colonization rather than pneumonia. However, often the challenge is not to discriminate between patients with pneumonia and those with positive cultures without pneumonia: in this study that difference is defined by the presence of symptoms and an inflammatory response. Consequently, 1-propanol can still be a marker of pneumonia in another scenario; when discriminating between patients with symptoms and positive cultures and patients with symptoms and negative cultures. Remarkably, more VOCs were significantly different between patients with and without colonized airways and the amount was higher than the amount of VOCs that distinguished pneumonia from controls. Furthermore, the majority of the relevant VOCs related to pneumonia had an AUROC above 0.7, while the majority of the VOCs related to finding the colonization status had an AUROC of less than 0.7. Thus, the significantly-altered VOCs related to pneumonia were stronger predictors. VOC formation and depletion have a complicated balance [32]. The relative composition of VOCs in exhaled air can change as a result of a disease that may lead to a decrease or an increase of a certain compound. VOCs could be produced by the host or by the bacteria. We hypothesize that in patients with a colonized respiratory tract the signal is predominantly altered by the bacteria while, in investigating pneumonia, the signal is also influenced by host-response. That these two processes contribute to changes in exhaled breath VOCs has been nicely demonstrated in animal studies [33].

The predicted probability for having pneumonia for patients that had colonized airways without pneumonia or had a possible pneumonia were in between the values that were found for the control group and patients with a probable pneumonia. This result was expected, because controls and patients with probable pneumonia represented the extremes in the spectrum of pneumonia, the remaining patients exemplified as subjects lying somewhere in between these two extremes. This finding emphasizes the plausibility of results.

Our study had several limitations. The most important limitation of this study was the small study group and a large amount of variables. This causes an increased likelihood of over-fitting [34]. Ideally, a training dataset was used to train the algorithm, which was validated with a test dataset [32,34]. In our study this was not possible due to the small number of patients with pneumonia. As second best, permutation tests and leave-one-out cross-validation were used. However, future studies should focus on validating this model in an independent group of subjects. External validation is not only of great significance for a statistical model in general, but also a key step in showing the superior diagnostic value of breath analysis in relation to other biomarkers in particular. Secondly, patients with and without airway colonization were not two separate clean groups. Microbiological cultures are not 100% sensitive nor specific and are, thus, an imperfect gold standard [5,35]. All patients without positive cultures were classified as non-colonized. This included patients with a high probability for pneumonia, but without proven causative agents by a positive culture. It is plausible that these cultures were falsely negative. Additionally, cultures in patients with a possible pneumonia could also be falsely negative. Breath profiles from these patients might interfere with the reported results. Some of the identified VOCs were of certain exogenous origin (the fluor-containing compounds, such as sevoflurane). It is very difficult to link these VOCs to pathophysiological processes that are associated with pneumonia. We, therefore, consider these VOCs as contamination and false-discovery. Nevertheless, these VOCs are included in the model due to their statistical significance. Due to the small sample size and the explorative nature of the study we were not able to combine the predictive value of the VOCs with that of clinical markers. Another point of discussion is the air used for sampling. Some authors have argued that expiratory alveolar air is the most appropriate fraction of breath to analyse [15]. We sampled a mixture of alveolar and dead space air. This method was chosen because it is a safe, non-invasive method that is easy to perform [36]. Breath was collected in tubes, which were connected for ten minutes to the circulation circuit. We assume this is sufficient to collect most VOCs in exhaled breath. Furthermore, in the control group significantly fewer patients were diagnosed with ARDS. Previous studies showed that ARDS results in altered breath profiles [37]. It is unclear how the unequal distribution of patients with ARDS influenced our results, although it should be noted that none of the identified VOCs were predictive of ARDS in a previous study [26]. One of the strengths of the study is that we did not only compare patients with pneumonia to controls but we also compared colonized and non-colonized patients. There is a clinically-relevant difference between merely the presence of bacteria versus the presence of bacteria that actually leads to infection. We were able to see that different VOCs discriminate between these conditions. Another strength is the group selection we used for building the classification model for predicting the probability for pneumonia. Only patients with a high suspicion or without any suspicion for respiratory tract infections were used to train the algorithm. Due to the lack of a good gold standard, two clinically well-defined groups were needed to determine reliably the accuracy of this new diagnostic test. Another strength is that the accuracy of the model was assessed with the AUROC as a measure of accuracy, which is proven suitable in classifying patients [38,39].

GC-MS analysis is impractical as a method for VOC detection in clinical practice; specialized employees are required, it is not available at the bedside, and the subsequent analysis is time-consuming. However, GC-MS is currently considered the gold standard for identifying distinct VOCs [15]. The knowledge provided in this paper may be utilized to develop a method that can rapidly detect the described VOCs at the bed-side in order to accurately diagnose or exclude pneumonia. Using a bedside sensor is non-invasive, fast, and completely safe [40,41].

## 4. Materials and Methods

### 4.1. Design, Subjects, and Setting

This was a prospective, single-centre, cross-sectional cohort study. The single inclusion criterion was an expected duration of mechanical ventilation of more than 24 hours. Cardiopulmonary surgery patients and patients that previously received mechanical ventilation in the same hospital admission were excluded. Exhaled air was sampled within 24 hours after ICU admission. Ethical approval was obtained from the institutional review board of the Academic Medical Centre, Amsterdam, the Netherlands. It was judged by the institutional review board that exhaled breath could be obtained and analysed without informed consent of the patient. The study was registered at the Dutch Trial Register (NTR 2750, available at: www.trialregister.nl).

### 4.2. Clinical Diagnosis of Pneumonia

A team of trained clinical research fellows prospectively scored the presence of pneumonia based on adapted Centre for Disease Control-criteria and a post-hoc likelihood of infection was scored (none, possible, probable, or definite; see Table 3 and Table 4). All assessors had attended meetings in which clinical case vignettes were discussed and had at least six months of work experience. The reliability of the diagnosis made by the team of researchers was evaluated and the results were published [42]. Endotracheal aspiration and culture were performed in all patients as surveillance cultures as per protocol required for the selective decontamination of the digestive tract regimen that is standard of care in the ICU where the study was performed [43,44]. Additional microbiological tests were performed on indication. 

### 4.3. Exhaled Breath Analysis and Data Pre-Processing

Exhaled breath was sampled and analysed by standardised methodology that was previously published [36]. In short, breath was collected through a disposable side-stream connection for 10 min and VOCs were stored on a sorbent tube. These tubes were analysed by means of thermal desorption GC-MS. Ion-fragments were detected and retention time correction was performed with the xcms package in the R environment (The R Foundation, Lucent Technologies, Inc., Murray Hill, NJ, USA) [45]. Ion counts of fragments within a small window of retention times (± 3 s) were summed to obtain a total ion count, if they strongly correlated (loaded onto the same principal component), in order to limit collinearity of the predictor matrix (e.g., to get one intensity per patient per VOC), but still allow for differentiation between co-elutions.

### 4.4. Group Comparisons and Analysis Plan

Patients with probable/proven pneumonia were considered cases, while patients without any signs of pneumonia and without colonized airways were considered controls. As the clinical diagnosis of pneumonia is a moderate gold standard, this approach allows separation of two clean groups at the extremes of the spectrum of disease. Significantly different VOCs were identified and the chances of false-discovery were calculated. Partial least square discriminant analysis was used to classify cases and controls. The over-optimism of the classifier due to over-fitting was estimated by leave-one-out cross-validation. The predicted probability for pneumonia was extrapolated to patients with a low likelihood for pneumonia and to patients without pneumonia with colonized airways. These steps of comparisons were repeated for patients with and without colonized airways, irrespective of the clinical suspicion of pneumonia.

### 4.5. Data Analysis 

All analyses were performed in R statistics (version 3.2.5) using the R-studio interface (The R Foundation, Lucent Technologies, Inc., Murray Hill, NJ, USA, www.r-project.org). Differences between the groups were compared using the Wilcoxon sum-rank test or Kruskal-Wallis test for continuous variables and Chi-squared for categorical variables. Data are presented as median (interquartile range) for skewed variables and *n* (%) for categorical variables.

Wilcoxon sum-rank tests were used to identify the VOCs that were significantly different between cases and controls. *p* values below 0.05 were considered to be statistically significant. Furthermore, fold changes between cases and controls were calculated. Area under the receiver operating characteristic curve (AUROC) was calculated with the pROC package in the R environment [46].

These results were visualized in a volcano plot. Due to the danger of false-discovery, all previous steps were repeated with 1000 datasets with permutated labels and the number of random scenarios that would result in a similar or better performance was calculated. Principal component analysis was performed to evaluate difference in breathprints between the groups. 

Partial least square discriminant analysis was performed to classify cases and controls. The predicted probability for pneumonia was calculated for each patient. Leave-one-out cross-validation was used to predict the accuracy of the models. This validation consists of a few steps. First one patient was excluded, and then the significant VOCs were selected to determine which data will be used to train the model. Afterwards, this model was used to calculate the predicted probability for pneumonia of the excluded patient. This procedure was repeated until every patient is once excluded. The AUROC was calculated for the discrimination between cases and controls. Additionally, the predicted probability was visualized between the four groups (cases, controls, and the two intermediate groups).

The statistical procedure described above was repeated for patients with and without colonized airways, irrespective of the clinical suspicion of pneumonia.

## Figures and Tables

**Table 1:** Patient demographics and clinical characteristics; data are presented as median (interquartile range) or *n* (%).

Clinical Data	Control *n* = 47	Colonization *n* = 13	Possible Pneumonia *n* = 21	Probable Pneumonia *n* = 12	*p*-Value
Age at ICU admission	59 (48–70)	64 (43–79)	63 (55–71)	61 (45–72)	0.93
Patient gender:					
Female	16 (34)	5 (38)	6 (29)	7 (58)	0.41
Male	28 (59)	8 (62)	15 (71)	5 (42)	
Admission type:					
Medical	31 (65)	8 (62)	20 (95)	11 (92)	0.17
Surgical elective	1 (2)	0 (0)	0 (0)	0 (0)	
Surgical emergency	12 (25)	5 (38)	1 (5)	1 (8)	
ICU Length of stay (days)	3 (2–5)	3 (2–4)	4 (3–5)	5.5 (3–9)	0.18
APACHE IV Score	80 (55–97)	76 (56–89)	76.5 (57–103)	66 (59–83)	0.74
ICU mortality	11 (23)	1 (8)	2 (10)	4 (33)	0.20
ARDS	2 (4)	12 (92)	15 (71)	9 (75)	<0.001
Positive Cultures	0 (0)	13 (100)	3 (14)	9 (75)	<0.001
Comorbidity:					
Malignancy	4 (9)	3 (23)	4 (19)	4 (33)	0.18
Diabetes Mellitus type 2	4 (9)	3 (23)	2 (10)	2 (17)	0.55
COPD	1 (2)	0 (0)	4 (19)	1 (8)	0.054
Asthma	0 (0)	0 (0)	1 (5)	0 (0)	0.49
Other	1 (2)	0 (0)	1 (5)	1 (8)	0.72
Pmax cm H_2_O	17 (14–22)	16 (13–17)	21 (18–24)	25 (22–28)	0.004
Peep cm H_2_O	5 (5–5)	5 (5–5)	8 (5–10)	9.5 (5–10)	0.001
Tidal Volume mL	458 (391–525)	467 (448–581)	500 (383–576)	464 (409–575)	0.74
FiO_2_ %	40 (35–40)	35 (35–40)	40 (35–45)	45 (40–60)	0.024
PaO_2_ kPa	13.8 (12.2–17)	16.3 (13.7–24.2)	14.7 (12.4–17.7)	14.2 (10.9–19.0)	0.31
PaCO_2_ kPa	5.1 (4.5–5.6)	5.1 (4.6–5.4)	5.5 (4.7–5.7)	5.1 (4.5–6.1)	0.58

**Table 2:** 2 × 2 tables. The partial least squares discriminant analysis (PLSDA) model was trained with significant volatile organic compounds (VOCs).

In-Set Analysis	Groups	Probable Pneumonia	Control
	**Probable pneumonia**	5	3
	**Control**	7	44
Leave-one-out validation		**Probable pneumonia**	**Control**
	**Probable pneumonia**	3	4
	**Control**	9	43
In-set analysis		**Positive culture**	**Negative culture**
	**Positive culture**	7	5
	**Negative culture**	18	63
Leave-one-out validation		**Positive culture**	**Negative culture**
	**Positive culture**	5	6
	**Negative culture**	20	62

**Table 3:** Adapted Centre for Disease Control-criteria for the likelihood of Community Acquired Pneumonia.

Community Acquired Pneumonia (*Symptoms of Pneumonia Started at Home or in First 48 h of Hospital Admission*)	Chest X-Ray and Clinical Suspicion	Symptoms
Possible	Uncertainty about infiltrates on chest X-ray	
	Low clinical suspicion, one or more of the following:	Cough
		Purulent sputum
		Fever or hypothermia
		Leukocytosis
		Increased C-reactive protein (CRP) (>30 mg/L)
		Hypoxia (pO_2_ < 60 mmHg)
Probable	Evident infiltrates on chest X-ray	
	High clinical suspicion, one or more of the following:	Crepitations during auscultation
		Positive pneumococcal or legionella urine test
Definite	Evident infiltrates on chest X-ray	
	High clinical suspicion	
	Causative organism detected, one or more of the following:	Positive blood culture
		High growth in tracheal aspirate
		Isolation of virus
		Positive serology
		Histopathology

**Table 4:** Adapted Centre for Disease Control-criteria for the likelihood of Hospital Acquired Pneumonia.

Hospital Acquired Pneumonia (*Symptoms of Pneumonia Started after 48h of Hospital Admission*)	Chest X-Ray and Clinical Suspicion		Symptoms
Possible	Uncertainty about infiltrates on chest X-ray		
	Low clinical suspicion, one or more of the following:	Cough	
		Purulent sputum	
		Fever or hypothermia	
		Leukocytosis	
		Increased CRP (>30 mg/L)	
		Hypoxia (pO_2_ < 60 mmHg)	
Probable	Evident infiltrates on chest X-ray		
	High clinical suspicion, one or more of the following:	Crepitations during auscultation	
		PaO_2_/FiO_2_ ratio <300	
		Mechanical ventilation	
	Causative organism detected, one or more of the following:	Detection of pathogen in respiratory secretion	
		Quantitative culture of bronchoalveolar lavage (BAL) / protected specimen brush (PSB) but below threshold for definite	
Definite	Evident infiltrates on chest X-ray		
	High clinical suspicion with one or more of the following:	Crepitations during auscultation	
		PaO_2_/FiO_2_ ratio <300	
		Mechanical ventilation	
	Causative organism detected, one or more of the following:	Positive blood culture with respiratory pathogen	
		Quantitative culture of BAL/PSB but above threshold (10^−3^ for PSB and 10^−4^ for BAL)	
		Isolation of virus	
		Positive serology	
		Histopathology	

## Abbreviations

ARDS	Acute Respiratory Distress Syndrome
AUROC	Area under the receiver operating characteristics curve
BAL	Bronchoalveolar lavage
CAP	Community-acquired pneumonia
CI	Confidence interval
COPD	Chronic Obstructive Pulmonary Disease
HAP	Hospital-acquired pneumonia
ICU	Intensive care unit
TD-GC-MS	Thermal desorption-gas chromatography-mass spectrometry
PC	Principal component
PLSDA	Partial least squares discriminant analysis
PSB	Protected specimen brush
VAP	Ventilator-associated pneumonia
VOC	Volatile organic compound
